# Doxorubicin-induced elevated oxidative stress and neurochemical alterations in brain and cognitive decline: protection by MESNA and insights into mechanisms of chemotherapy-induced cognitive impairment (“chemobrain”)

**DOI:** 10.18632/oncotarget.25718

**Published:** 2018-07-13

**Authors:** Jeriel T. R. Keeney, Xiaojia Ren, Govind Warrier, Teresa Noel, David K. Powell, Jennifer M. Brelsfoard, Rukhsana Sultana, Kathryn E. Saatman, Daret K. St. Clair, D. Allan Butterfield

**Affiliations:** ^1^ Department of Chemistry, University of Kentucky, Lexington, KY 40506, USA; ^2^ Department of Toxicology and Cancer Biology, University of Kentucky, Lexington, KY 40536, USA; ^3^ Magnetic Resonance Imaging and Spectroscopy Center, University of Kentucky Medical Center, Lexington, KY 40536, USA; ^4^ Spinal Cord and Brain Injury Research Center, University of Kentucky, Lexington, KY 40536, USA; ^5^ Department of Radiation Medicine, University of Kentucky, Lexington, KY 40502, USA; ^6^ Markey Cancer Center, University of Kentucky, Lexington, KY 40502, USA; ^7^ Sanders Brown Center on Aging, University of Kentucky, Lexington, KY 40536, USA

**Keywords:** chemotherapy induced cognitive impairment, oxidative stress, choline, cognitive dysfunction

## Abstract

Chemotherapy-induced cognitive impairment (CICI) is now widely recognized as a real and too common complication of cancer chemotherapy experienced by an ever-growing number of cancer survivors. Previously, we reported that doxorubicin (Dox), a prototypical reactive oxygen species (ROS)-producing anti-cancer drug, results in oxidation of plasma proteins, including apolipoprotein A-I (ApoA-I) leading to tumor necrosis factor-alpha (TNF-α)-mediated oxidative stress in plasma and brain. We also reported that co-administration of the antioxidant drug, 2-mercaptoethane sulfonate sodium (MESNA), prevents Dox-induced protein oxidation and subsequent TNF-α elevation in plasma. In this study, we measured oxidative stress in both brain and plasma of Dox-treated mice both with and without MESNA. MESNA ameliorated Dox-induced oxidative protein damage in plasma, confirming our prior studies, and in a new finding led to decreased oxidative stress in brain. This study also provides further functional and biochemical evidence of the mechanisms of CICI. Using novel object recognition (NOR), we demonstrated the Dox administration resulted in memory deficits, an effect that was rescued by MESNA. Using hydrogen magnetic resonance imaging spectroscopy (H^1^-MRS) techniques, we demonstrated that Dox administration led to a dramatic decrease in choline-containing compounds assessed by (Cho)/creatine ratios in the hippocampus in mice. To better elucidate a potential mechanism for this MRS observation, we tested the activities of the phospholipase enzymes known to act on phosphatidylcholine (PtdCho), a key component of phospholipid membranes and a source of choline for the neurotransmitter, acetylcholine (ACh). The activities of both phosphatidylcholine-specific phospholipase C (PC-PLC) and phospholipase D were severely diminished following Dox administration. The activity of PC-PLC was preserved when MESNA was co-administered with Dox; however, PLD activity was not protected. This study is the first to demonstrate the protective effects of MESNA on Dox-related protein oxidation, cognitive decline, phosphocholine (PCho) levels, and PC-PLC activity in brain and suggests novel potential therapeutic targets and strategies to mitigate CICI.

## INTRODUCTION

Chemotherapy-induced cognitive impairment (CICI), often termed “chemobrain” by patients, is increasingly recognized as a significant complication of cancer chemotherapy [1–7]. CICI consists of impairments in various aspects of memory and executive function [8, 9]. Despite the increased attention this issue has garnered from the clinical and research communities, the mechanisms of the resulting cognitive impairment still are poorly understood but are thought to include peripheral toxic effects caused by the chemotherapy drugs leading to downstream structural and functional changes in the brain. These latter changes include neuroinflammatory consequences and even changes in neurotransmitter levels and function [10–13]. The main reasons for the slowness to address CICI may include the complexity of cancer and its treatments, especially by agents that do not cross the blood-brain barrier (BBB). Moreover, a hitherto lack of a scientific explanation for cognitive consequences of chemotherapy has hampered progress. A better understanding of the underlying mechanisms by which CICI occurs is necessary to allow cancer survivors to have a better quality of life by protecting non-targeted tissues against undesired toxicities of anticancer drugs.

In the present studies, we used doxorubicin (Dox) as a representative chemotherapeutic agent known to produce reactive oxygen species (ROS) [14–16]. Dox is an anthracycline antineoplastic agent commonly used in multidrug chemotherapy regimens primarily to treat solid tumors and leukemia. The cancer-killing effects of Dox have been shown to involve three proposed mechanisms: DNA intercalation, inhibition of topoisomerase II, and production of ROS [16–22]. The quinone moiety present in the Dox structure is capable of undergoing a one-electron reduction to the semi-quinone [10, 23]. Through the redox cycling of this structure back to the quinone *in vivo*, the reactive superoxide free radical (O_2_**^-^**·) is produced from molecular oxygen. In addition, previous studies by our laboratories demonstrated that even though neither Dox nor its primary metabolite crosses the BBB, peripheral Dox treatment causes brain injury as evidenced by increased oxidative stress, elevated levels of the pro-inflammatory cytokine, tumor necrosis factor-alpha (TNF-α), and mitochondrial dysfunction [24–27].

Our laboratory and others previously demonstrated Dox-induced oxidative stress in plasma and damage to plasma proteins subsequently leading to detrimental central nervous system consequences [10, 24, 28–31]. Central to this paradigm is apolipoprotein A-I (ApoA-I) [4, 10, 32, 33]. ApoA-I promotes cholesterol efflux as part of the high density lipoprotein (HDL) complex. Additionally, ApoA-I has been shown to suppress TNF-α in plasma [34–36]. Previous studies showed that, when oxidized, ApoA-I loses this ability to suppress TNF-α release and may exacerbate the problem [10].

However, ApoA-I oxidation and subsequent increased TNF-α release is suppressed with co-administration of the drug MESNA (2-mercaptoethane sulfonate sodium) in mice [10]. The structure of MESNA contains a free sulfhydryl group imparting much of its antioxidant properties by affording it the ability to scavenge free radicals and lipid-derived reactive aldehydes such as 4-hydroxynonenal (HNE) and acrolein. MESNA is FDA approved for prevention of hemorrhagic cystitis and routinely used with Dox as part of multidrug chemotherapy regimens that include ifosfamide or cyclophosphamide. MESNA does not enter cells and therefore does not interfere with cancer chemotherapy [37]. Treatment of mice with MESNA blocks protein oxidation, including ApoA-I, in the plasma [10]. Modulating the location and production of chemotherapy-induced production of ROS may be paramount in decreasing the unwanted toxicities associated with chemotherapy while enhancing the cancer-killing effects [38].

The current study was undertaken to test the hypothesis that MESNA would block Dox-induced, TNF-α-mediated markers of brain damage, indexed by changes in oxidative stress and magnetic resonance spectroscopy (MRS) spectra in brain, with consequent improved cognition.

## RESULTS

### Dox administration results in increases in oxidative stress markers in brain and plasma

**Figure 1:**
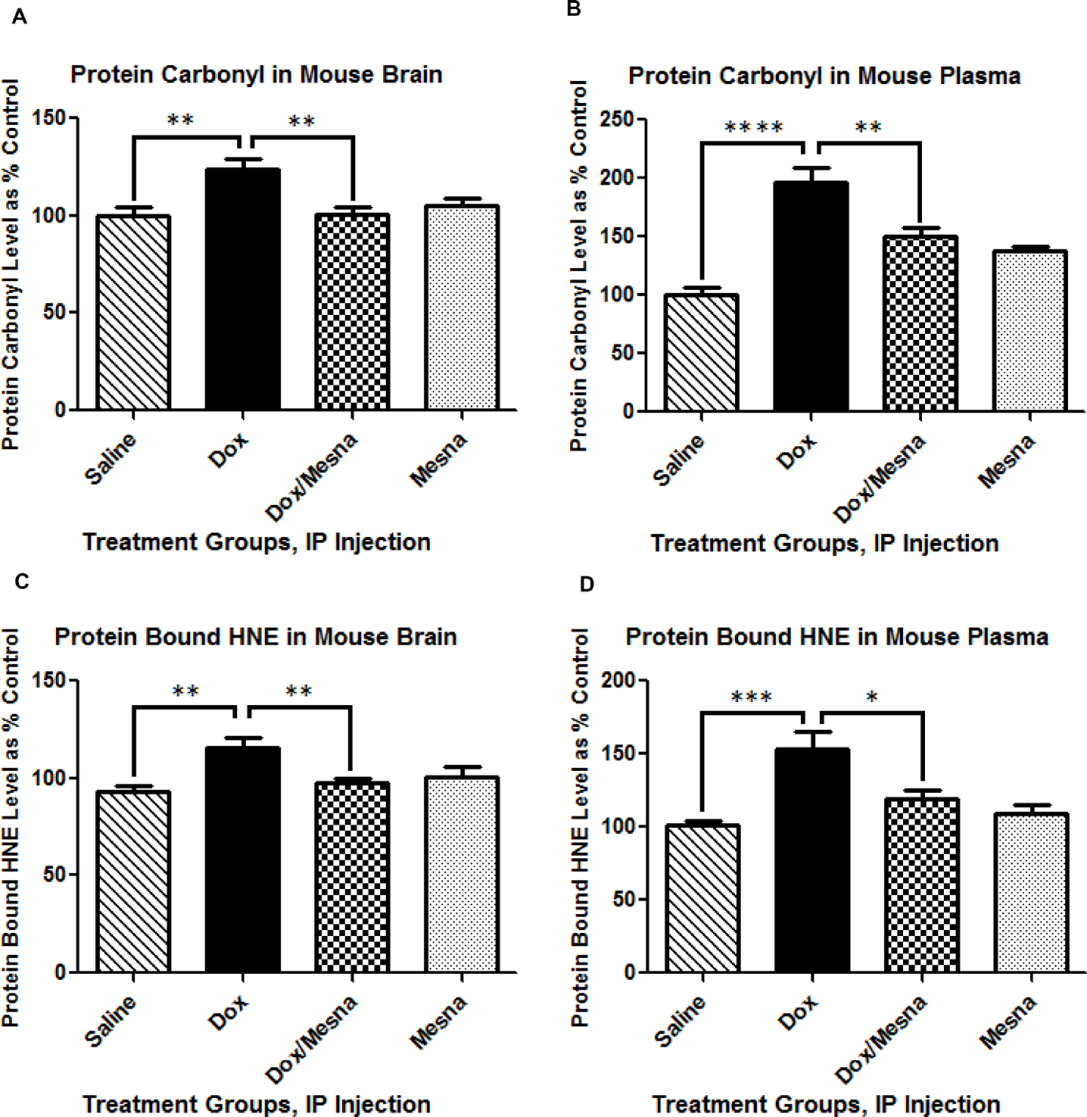
Levels of protein carbonyl and protein-bound HNE are indicators of protein oxidation and lipid peroxidation, respectively Graphs (**A**–**D**) depict protein carbonyl and protein-bound HNE in brain and plasma of 2–3 month old, male B6C3 mice treated with saline, MESNA, Dox (25 mg/kg), or Dox with MESNA. MESNA was administered at 160 mg/kg i.p. 15 min before DOX/Saline as well as 3 h and 6 h after Dox/Saline. Brain and plasma samples were acquired post 72 h treatments. Protein carbonyl levels were significantly increased in brain (A) (^**^P < 0.01) and plasma (B) (^****^P < 0.0001) of mice treated with Dox relative to saline. MESNA, co-administered with Dox, ameliorated Dox-induced increases in protein carbonyl in both brain and plasma (^**^P < 0.01). Protein-bound HNE levels were significantly elevated in brain (^**^P < 0.01) and plasma (^***^P < 0.001) of mice treated with Dox relative to saline. MESNA, co-administered with Dox, significantly suppressed Dox-induced elevation in protein-bound HNE in both brain (^**^P < 0.01) and plasma (^*^P < 0.05). *N* = 10–13 per treatment group.

We previously showed that, despite its inability to cross the BBB, peripheral Dox administration led to increased levels of TNF-α and oxidative stress in brain [10, 11, 24, 27, 39]. Here, we tested for indicators of oxidative stress in brain and plasma of animals used in this study. Test subjects were administered either saline, MESNA, Dox, or Dox plus MESNA. Brain and blood samples were collected 72 h post-Dox treatment, immediately following cognitive or MRS studies. Protein carbonyl and protein-bound HNE levels were used as a gauge of damage to proteins and lipids, respectively. Significantly higher levels of protein carbonyls and protein-bound HNE in brain of Dox-treated animals compared to saline-treated controls were observed (*p <* 0.01, Figure 1A and *p* < 0.01, Figure 1C, respectively). MESNA protected the brain from these oxidative damages (*p <* 0.01, *p <* 0.01). Plasma results confirmed similarly increased protein carbonyl and protein-bound HNE levels in Dox vs. saline treated animals (*p <* 0.0001, Figure 1B and *p* < 0.001, Figure 1D, respectively). Both effects were ameliorated when MESNA was administered with Dox (*p <* 0.01, *p <* 0.05). These results are consistent with our previous findings in plasma [10] and brain [11, 27] and consistent with the notion that concomitant MESNA administration may be able to reduce or prevent these consequences in brain.

### Dox administration results in cognitive impairment and decreased locomotor activity

**Figure 2:**
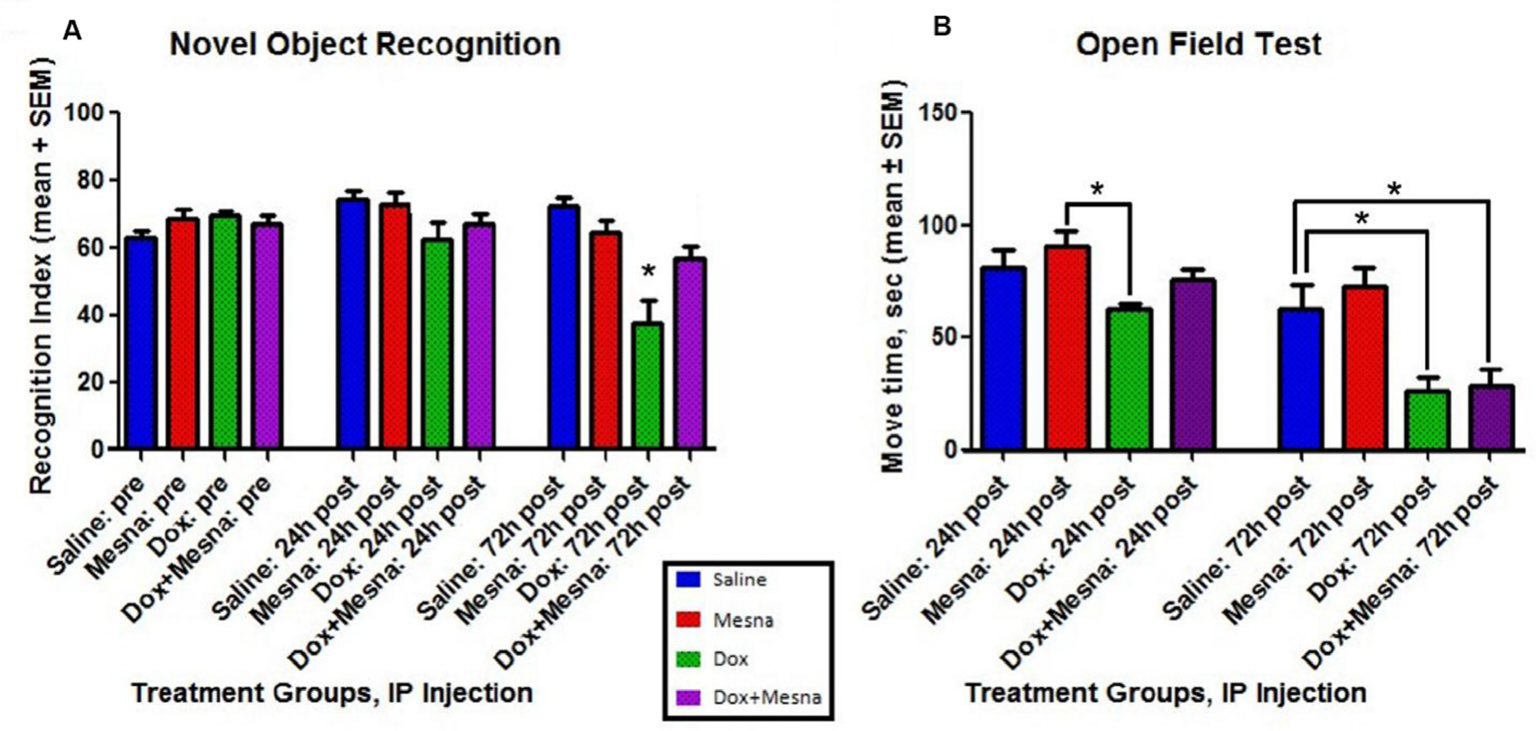
Behavior testing for male B6C3 mice treated with saline, MESNA, Dox (25 mg/kg), or Dox+MESNA MESNA was administered at 160 mg/kg i.p. 15 min before DOX/ Saline as well as 3 h and 6 h after Dox/Saline. Brain and plasma samples were acquired post 72 h treatments. *N* = 7–8 per treatment group. (**A**) Novel Object Recognition (NOR) testing provides a measure of cognitive function through recognition memory. Dox group performed at a significantly lower RI than Saline, MESNA and Dox+MESNA groups at 72 h post treatment (^*^P < 0.05). MESNA given with Dox rescued this measure of cognitive function. (**B**) Open Field Testing was used to provide a comparison of average total locomotor activity among groups. Dox group is significantly different from MESNA control group at 24 h post treatment. Both the Dox and Dox+MESNA groups showed significantly decreased average total movement compared to Saline group or MESNA group at 72 h post treatment (^*^P < 0.05). Comparisons to MESNA group at 72 h were not shown in the figure. The result shows that motor activity in an open field declines following Dox treatment, and this motor dysfunction is not ameliorated by MESNA treatment.

In order to determine potential cognitive consequences of Dox-induced oxidative stress in brain and the possibility that MESNA protected brain function, NOR was performed on animals in each of the previously mentioned treatment groups (Figure 2A). NOR provides a measure of cognitive function in rodent models by assessing the preference for investigating a novel object in a familiar environment. Preference for the novel object indicates memory of a familiar object and learning through the animal's natural propensity to explore an unfamiliar object. Novelty recognition is thought to require more complex cognitive function [40, 41]. NOR employs both hippocampus and frontal cortex, thought to be key brain regions affected in CICI (“chemobrain”). Open field testing was employed as a measure of locomotor activity among treatment groups (Figure 2B).

Prior to treatment, animals assigned to each of the treatment groups spent an average of 65–70% of total exploration time investigating the novel object. At 24 h post-treatment, each subject was re-acclimated to the environment containing the two original familiar objects followed by exposure to one familiar and one novel object. There is no difference among each treatment groups at 24 h.

At 72 h post-treatment, the saline and MESNA treatment groups maintained an average RI of approximately 70, similar to previous performance. Meanwhile, the Dox treatment group performed at a significantly decreased mean RI of around 40, suggesting no preference for the novel object, reflecting Dox-induced memory impairment. The RI of Dox treated group at 72 h post-treatment was significantly lower than all three other groups (Saline, MESNA and Dox+MESNA) at 72 h post-treatment (^*^P < 0.05), suggesting a delayed memorydecline by Dox. That corresponded to the time when oxidative stress parameters were significantly elevated (Figure 1). The group that received MESNA with Dox had a mean RI of approximately 60 at 72 h post-treatment, significantly higher than the 72 h Dox treated group, indicating a protection of cognitive function by rescuing the Dox-induced delayed memory impairment.

Dox treatment significantly decreased the locomotor activity compared to MESNA control group at 24 h post treatment (^*^P < 0.05). Dox resulted in a progressive decline in locomotor activity, which reached statistical significance by 72 h post-treatment in Figure 2B (^*^P < 0.05). The similar decrease in total locomotor activity of the Dox and Dox+MESNA groups (Figure 2B) suggests a selective effect of MESNA on attenuating Dox-induced memory impairment, and eliminates activity level as a potential confounding variable when comparing cognitive function results between these two groups.

### Dox administration results in changes to the neurochemical profile in the hippocampus determined by MRS

**Figure 3:**
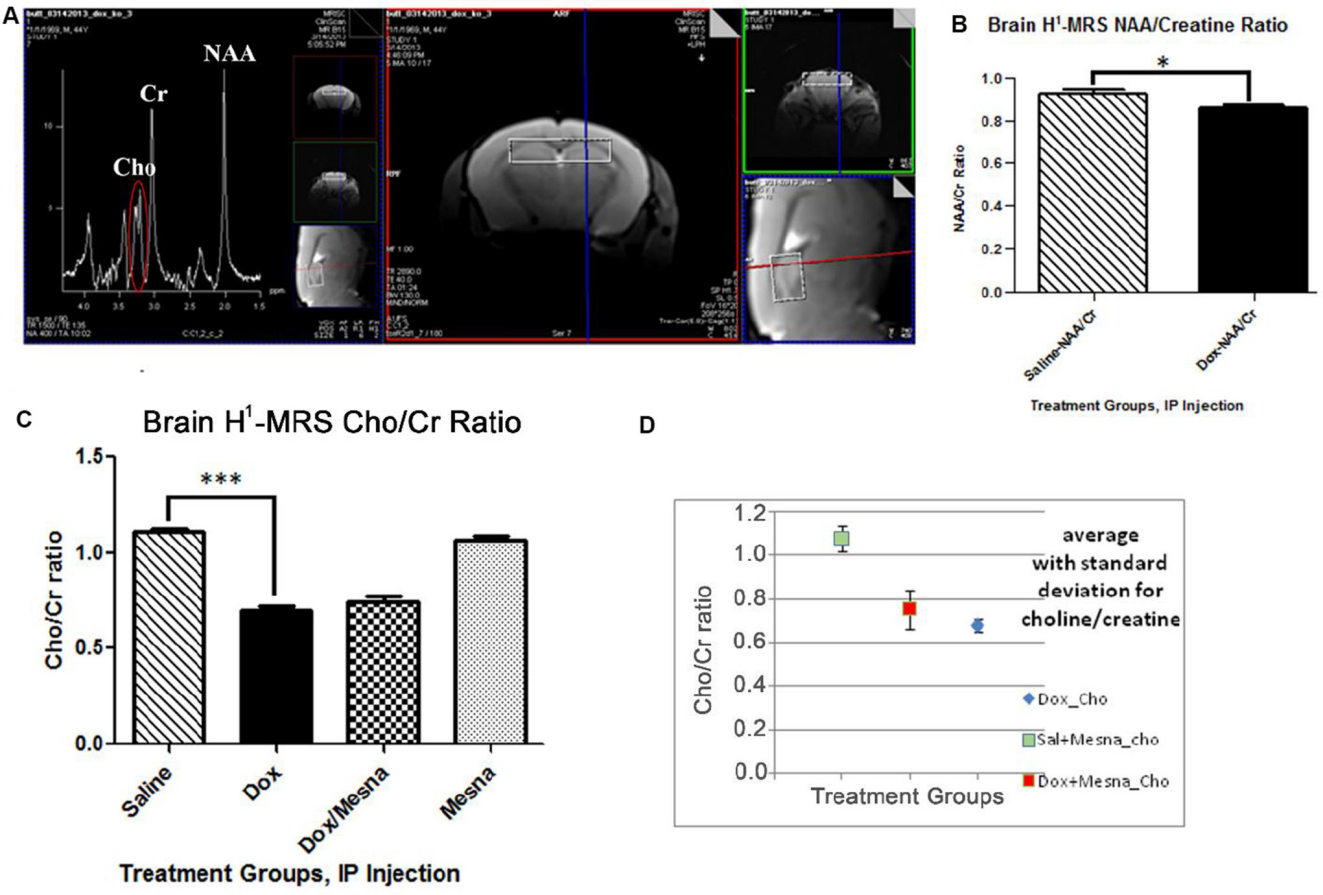
(**A**) H^1^-MRS uses proton signatures from hydrogen in much the same way as NMR to create two-dimensional images of the tissue (right) and a spectrum of peaks reflecting a neurochemical profile of one mouse after 72 h treatment that includes NAA, Cho, Cr, and others (left). (**B**) Bilateral H^1^-MRS scans of mouse hippocampus revealed that Dox treatment lead to a slight but statistically significant decrease compared to Saline group (^*^P < 0.05) in the NAA/Cr ratio. (**C**) A six standard deviation decrease in the Cho/Cr ratio in the Dox-treated group compared to saline control (^***^P < 0.001). (**D**) Co-administration of MESNA with Dox resulted in a trend toward rebound in Cho/Cr compared to Dox alone. Greater variability was seen in Cho/Cr in the Dox+MESNA group.

The involvement of the hippocampus in learning and memory [40, 42–45] led us to pursue MRS scans of the hippocampus in the murine chemotherapy treatment groups studied. H^1^-MRS non-invasively measures neurochemical aspects of the living brain. The peaks observed in this spectrum (Figure 3A) include N-acetylaspartate (NAA), Choline-containing compounds (Cho), creatine (Cr), myo-inositol, glutamate and glutamine, lipids, and lactate allowing quantification of these and other metabolites in the living brain [46]. Quantification is generally achieved using ratios to other species, commonly to Cr.

Unilateral and bilateral hippocampal H^1^-MRS showed a slight, but significant, decrease in the NAA/Cr ratio in the Dox treated group compared to saline controls (*p <* 0.05, Figure 3B). A decrease in NAA/Cr is indicative of decreased neuronal integrity. Strikingly, MRS scans revealed, on average, a much larger six standard-deviation decrease in Cho/Cr in Dox-treated mice compared to that of saline-treated mice (*p <* 0.0001, Figure 3C). Though not significant, a slight increase in the Cho/Cr to peak was seen in the Dox+MESNA group compared to Dox alone (Figure 3D). This result suggests that MESNA may be protecting cognition by a different mechanism in addition to partial restoration of the Cho/Cr ratio. As noted above, phosphocholine and glycerophosphorylcholine are the major contributors to the Cho peak, while choline itself is a smaller contributor.

### Dox administration results in decreased PC-PLC and PLD activity

**Figure 4:**
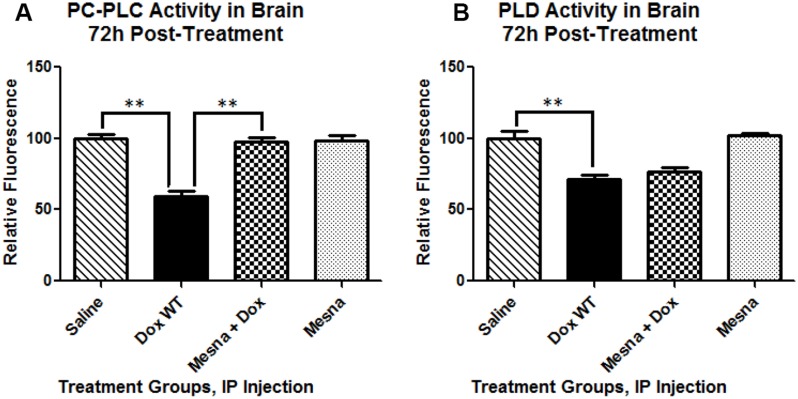
Phosphatidylcholine-specific phospholipase C (PC-PLC) and Phospholipase D (PLD) activity in brain 72 h post-treatment presented as percent saline control (**A**) PC-PLC activity at 22.5 h of incubation, the peak fluorescence of the corresponding positive control in these trials, at room temperature in the dark. Cleavage of the assay substrate by PC-PLC yields a dye-labeled diacylglycerol (DAG) which fluoresces using an excitation and emission maxima of 509 nm and 516 nm, respectively. Dox administration caused a significant decrease in PC-PLC activity compared to saline treated mice (^**^p < 0.01). Co-administration of MESNA rescued decreased PC-PLC activity by Dox (^**^p < 0.01). (**B**) PLD activity at 1h of incubation, the peak fluorescence of the corresponding positive control in these trials, at 37° C protected from light. PLD cleaves the headgroup from phospholipids thereby releasing the choline from PtdCho. Assay reactions involving the choline produce a product that fluoresces using an excitation and emission maxima of 571 and 585 nm, respectively. Dox treatment resulted in significantly decreased PLD activity compared to saline treated controls (^**^p < 0.01). PLD activity in the Dox+MESNA group was not significantly different from the group receiving Dox alone.

To gain insight into a possible mechanism for the MRS-indexed changes in choline-containing compounds, the activities of phospholipase enzymes known to act on PtdCho, a major source of choline and PCho in the brain were tested. PC-PLC cleaves PtdCho at the glycerol-phosphate bond producing the second messenger, diacylglycerol (DAG), and PCho. Phospholipase D (PLD), located in the plasma membrane, cleaves the headgroup from phospholipids thereby releasing soluble choline from PtdCho into the cytosol leaving phosphatidic acid. Activity of both PC-PLC and PLD in brain were severely impaired at 72 h following Dox administration (*p <* 0.01 and *p <* 0.01, respectively; Figure 4) providing a possible explanation for the dramatic decrease seen in the choline-containing peaks (as measured by MRS Cho/Cr ratio). MESNA, co-administered with Dox, completely rescued PC-PLC activity back to the activity observed in the saline-treated group. However, adding MESNA to the treatment regimen with Dox did not prevent the Dox-related decrease in PLD activity.

## DISCUSSION

**Figure 5:**
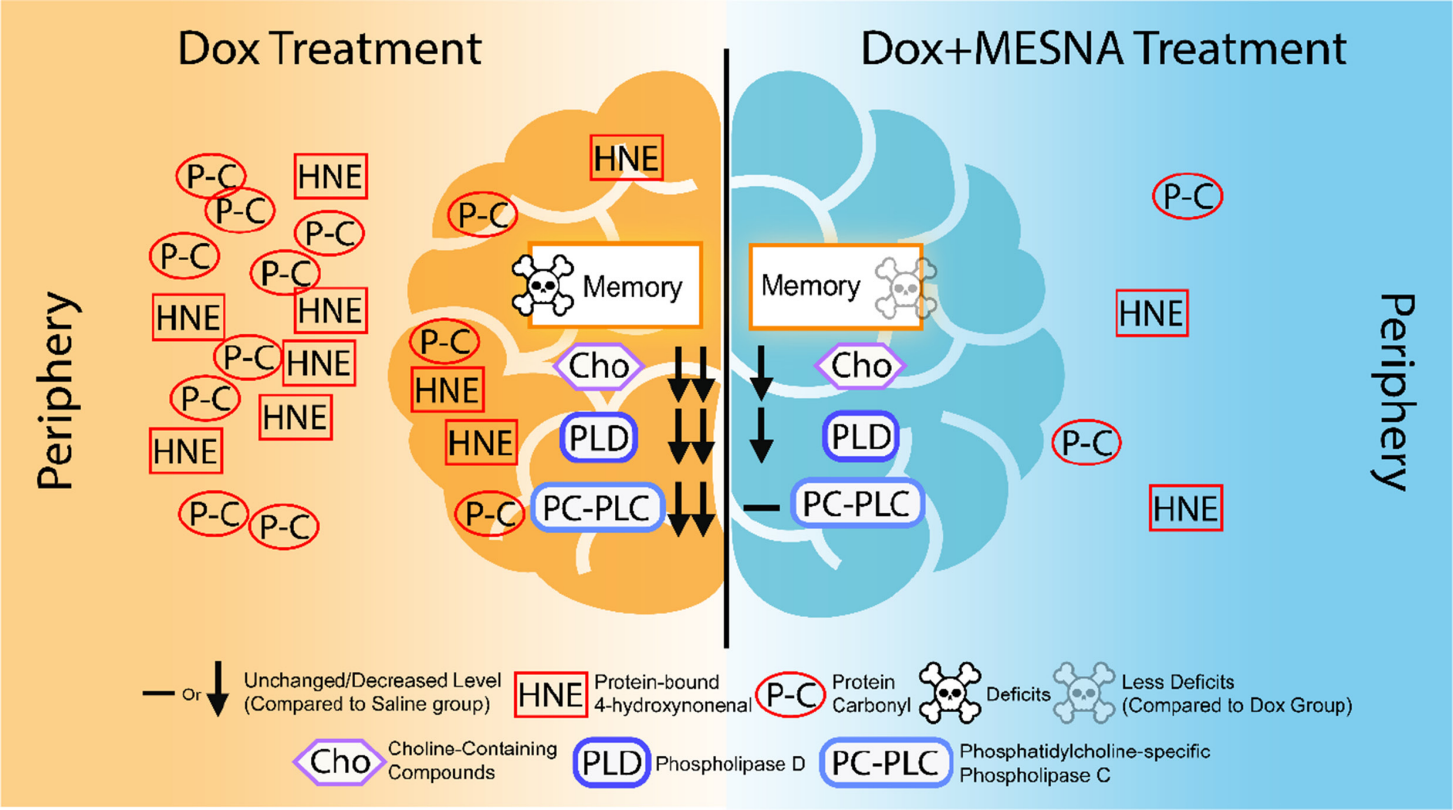
A pictorial summary of results of Dox-induced elevated oxidative stress and neurochemical alterations in the periphery and brain as well as cognitive decline (left) and MESNA-mediated protection against these Dox-facilitated effects in both plasma and brain

Based on our earlier studies, we previously proposed the following model for CICI [47]: Plasma protein oxidation, including that of ApoA-I, induced by the redox cycling of Dox, leads to elevation of TNF-α in the periphery. TNF-α crosses the BBB by receptor-mediated endocytosis to induce microglial activation, leading to further TNF-α release, increased production of NO, mitochondrial dysfunction, neuronal death, and consequent cognitive impairment [4, 10, 11, 24–26, 28]. We previously showed that oxidative damage occurs early in neurodegenerative processes [48–52]. Nearly half of FDA approved anti-cancer drugs result in elevation of ROS and induce oxidative stress [23]. A large percentage of cancer survivors suffer from CICI, now widely recognized as a chemotherapy complication [1–5]. The goals of this study were to gain insights into mechanisms of CICI and its potential prevention, with the long-term goal to progress toward prevention or at least successful management of CICI and an improved quality of life for an ever-growing number of cancer survivors. Figure 5 depicts major changes in brain following treatment of mice with non-BBB permeable Dox and modulation or amelioration of these changes by MESNA.

Direct toxic effects caused by the chemotherapy drugs lead to damage to biomolecules including lipids, proteins, lipoproteins, and genetic material [10, 11, 23, 53–55]. Oxidative stress caused by anticancer drugs leads to damage to biomolecules in non-targeted, non-cancerous tissues including the blood, heart, and brain [23, 47]. Reactive oxygen and reactive nitrogen species (ROS, RNS) include such species as superoxide radical anion (O_2_**^-^**·), nitric oxide (NO), peroxynitrite (ONOO**^-^**), and hydroxyl radical (HO·). Some of these have both functions essential to life and effects damaging to biomolecules necessary for life. O_2_**^-^**· is produced by inefficient reduction of molecular oxygen in the mitochondria [23, 56–58]. NO, a free radical, is produced from L-arginine by catalysis of various forms of the enzyme, nitric oxide synthase. Together, O_2_**^-^**· and NO combine to form another reactive species, ONOO^-^ [59, 60]. Hydrogen peroxide (H_2_O_2_), produced through the actions of superoxide dismutase (SOD) [61, 62], is converted to water and molecular oxygen by peroxidases, but H_2_O_2_ can result in production of HO· in the presence of iron(II) or copper(I) ions via Fenton Chemistry [63–65]. These radical species can cause the formation of carbon-centered radicals, alkoxyl radicals, and peroxyl radicals further damaging biomolecules through a free radical chain reaction [66, 67]. In particular, this type of oxidative stress can lead to lipid peroxidation in the lipid bilayer and the formation of reactive alkenals such as HNE, a lipid peroxidation product easily formed in brain containing abundant arachidonic acid. HNE can covalently bind proteins by Michael addition to alter protein structure and function [68–71]. The brain is particularly vulnerable to oxidative damage due to relatively low antioxidant defenses, high oxygen consumption, and high concentrations of polyunsaturated fatty acids.

Administration of the prototypical ROS-generating anti-cancer drug, Dox, leads to oxidative damage to plasma proteins through both direct and indirect toxicity independent of its cancer killing ability. Dox directly continually causes oxidative stress in peripheral tissues by redox cycling of the quinone moiety in its structure [72]. Dox-induced cardiac dysfunction, in part is due to mitochondrial damage, is well established and is used as a dose limiting criteria in treatment protocols [73–76]. Indirectly, Dox elevates levels of TNF-α in the plasma and, subsequently, the brain leading to neuronal death [10, 24]. Macrophages are the principal cell source of TNF-α; however, cellular targets and biological effects are varied including inflammation, neutrophil activation, catabolism in fat and muscle, triggering the synthesis of acute-phase proteins, and apoptosis in many cell types. Such responses can be beneficial if acute but quite harmful if chronic or sustained.

Under normal conditions, ApoA-I suppresses TNF-α release in plasma [34, 77]. Once oxidized, ApoA-I loses this ability and may actually exacerbate TNF-α release [10, 32]. The oxidative status of ApoA-I is crucial to its role in TNF-α suppression. Dox-induced ApoA-I oxidation and TNF-α increase is suppressed by co-administration of MESNA [10]. MESNA is rapidly oxidized, scavenging reactive species in circulation. MESNA's time in circulation is short-lived as it is rapidly renally eliminated, thereby reducing the chance for potential unwanted side effects [78].

Oxidative stress data presented here support the results of our previous studies. Dox administration leads to oxidative stress in both plasma and brain as evidenced by increased protein carbonyl and protein-bound HNE. Both of these damages are prevented when MESNA is administered just prior to Dox. These results, shown for the first time in brain, confirm our prior results in plasma.

Cognitive testing of saline, Dox, MESNA, and Dox+MESNA treatment groups revealed memory impairment in animals receiving Dox alone. Cognitive performance, as measured by NOR, was rescued in the group that received MESNA with Dox at 72 h post-treatment. Open field testing was employed as a gauge of locomotor activity. The treatment groups receiving Dox displayed less total movement than those without Dox treatment. This finding is consistent with prior studies of others [79, 80] that conceivably could be due to protein oxidation of muscles and/ or the effects of elevated levels of the pro-inflamatory cytokine TNF-α [81, 82]. Dox-induced motor dysfunction is not ameliorated by MESNA treatment. However, the Dox and Dox+MESNA treatment groups displayed similar total movement in the test environment decreasing potential confounds when comparing NOR performance between these two groups. Total object exploration time was similar among all treatment groups and decreased with repeated exposure to the environment. After only 24 h post-injection, animals in the Dox-treated group were already showing a trend of decreased preference for the novel object compared to the other treatment groups. By day three, the Dox treated animals on average displayed no preference for the novel object over the familiar one. This is compelling evidence for Dox-induced cognitive impairment. MESNA rescued much of this Dox-induced cognitive deficit (Figure 2A), which we speculate is due to prevention of oxidative stress in brain following Dox treatment (Figure 1).

H^1^-MRS of hippocampus of similarly treated animals revealed changes in the neurochemical profile in Dox-treated group versus saline control. A slight but significant decline in the NAA/Cr ratio was observed in the Dox group suggesting neuronal damage. More profoundly, MRS revealed, on average, a large six standard deviation decline in the Cho/Cr ratio in the hippocampus of the Dox-treated group compared to the saline-treated group. These results are consistent with results from another study in which Ciszkowska-Łysoń *et al*. observed a time-related decrease in the Cho/Cr ratio following chemotherapy which they attributed to potential myelin damage [83]. Three days after Dox treatment may be too soon to see measurable myelin damage in brain detectable by MRS.

Changes in choline-containing compounds on MRS are thought to be associated with membrane turnover (phospholipid synthesis and degradation) [84, 85]. Choline levels have been shown to be proportional to cell density [86] and to correlate with degree of malignancy in cancers [84, 85]. A decrease in the Cho peaks in brain also has been seen in brain aging as well as a decrease in choline uptake in older adults [85, 87, 88]. A decrease in the Cho/Cr ratio is indicative of decreased cell density and necrosis [85, 86]. Upon Dox administration, the decreased PCho and/or choline is consistent with changes in the production or metabolism of PtdCho or the neurotransmitter, acetylcholine (ACh) [89, 90]. Due to the widespread functions of ACh in both the motor and somatic divisions of the autonomic nervous system, the effects of chemotherapy-induced changes to this neurotransmitter may be varied and dramatic and need to be further explored. Evidence exists that ACh-associated memory, intelligence, and mood may, in part, be mediated by choline levels and ACh metabolism in the brain [91].

As noted, the decrease in the Cho peak in brain revealed by MRS spectra of Dox-treated mice may be due to decreased membrane synthesis, decreased myelination, and potentially cell loss. Such observed changes by MRS conceivably may be consistent with white matter changes seen in human breast cancer patients [92–95]. Magnetic resonance imaging (MRI) and magnetic resonance spectroscopy (MRS) are proving to be useful tools both for visualization of white matter changes and changes in the neurochemical profile indicative of axonal degeneration and demyelination in the brains of living subjects following chemotherapy [95]. Indeed, the integrity of lipid-rich myelin covered white matter have been shown to be altered as well as damage to gray matter with associated functional deficits following systemic cancer chemotherapy, in some cases years after chemotherapy [8, 95, 96]. Studies have shown that chemotherapy-induced neuroinflammation, including increases in TNF-α, are correlated with changes in myelination and cognitive impairment [96]. Coupled with neuropsychological tests, neuroimaging techniques can provide important information to help outline a mechanism for clinical and biochemical changes in the brain that results from chemotherapy and help researchers and clinicians work together to decrease or prevent unnecessary cognitive decline in cancer patients [97–99].

Elevated TNF-α is reported to decrease synthesis of PtdCho [90, 100]. We previously hypothesized that TNF-α elevated in the periphery and in brain following cancer chemotherapy plays a central role in CICI [4, 10, 24, 26, 39, 47, 101]. PtdCho is also the principal phospholipid found in high-density lipoprotein (HDL), teaming with ApoA-I in cholesterol transport, one of the most common constituents found in biological membranes. PtdCho is usually located in the outer bilayer leaflet with the choline head group exposed to the aqueous cytosolic environment playing vital roles in membrane-mediated cell signaling. The turnover of PtdCho is reportedly accelerated in models of mitochondrial dysfunction mimicking Alzheimer disease (AD) patterns of metabolic changes in the brain [102]. Cleavage of PtdCho by PC-PLC yields PCho and the second messenger DAG, whereas cleavage of PLD releases choline used in the synthesis of the neurotransmitter, ACh. PC-PLC plays vital roles in several cell signaling pathways involved in both apoptosis and cell survival and in a variety of disease processes. Hence, decreased DAG could severely impair normal cell signaling and be important for CICI. Dox-treatment severely impaired the activity of both PC-PLD and PLD in brain of the mouse model of chemotherapy used in this study (Figure 4). The decrease in activity of these two enzymes may, in part, explain the changes in the Cho signal seen on hippocampal H^1^-MRS. The slight though not statistically significant recovery of the Cho peaks when MESNA was co-administered with Dox may be due to protection of PC-PLC function by MESNA but no protection of PLD. Therefore, more studies are warranted to further elucidate this mechanism.

**Figure 6:**
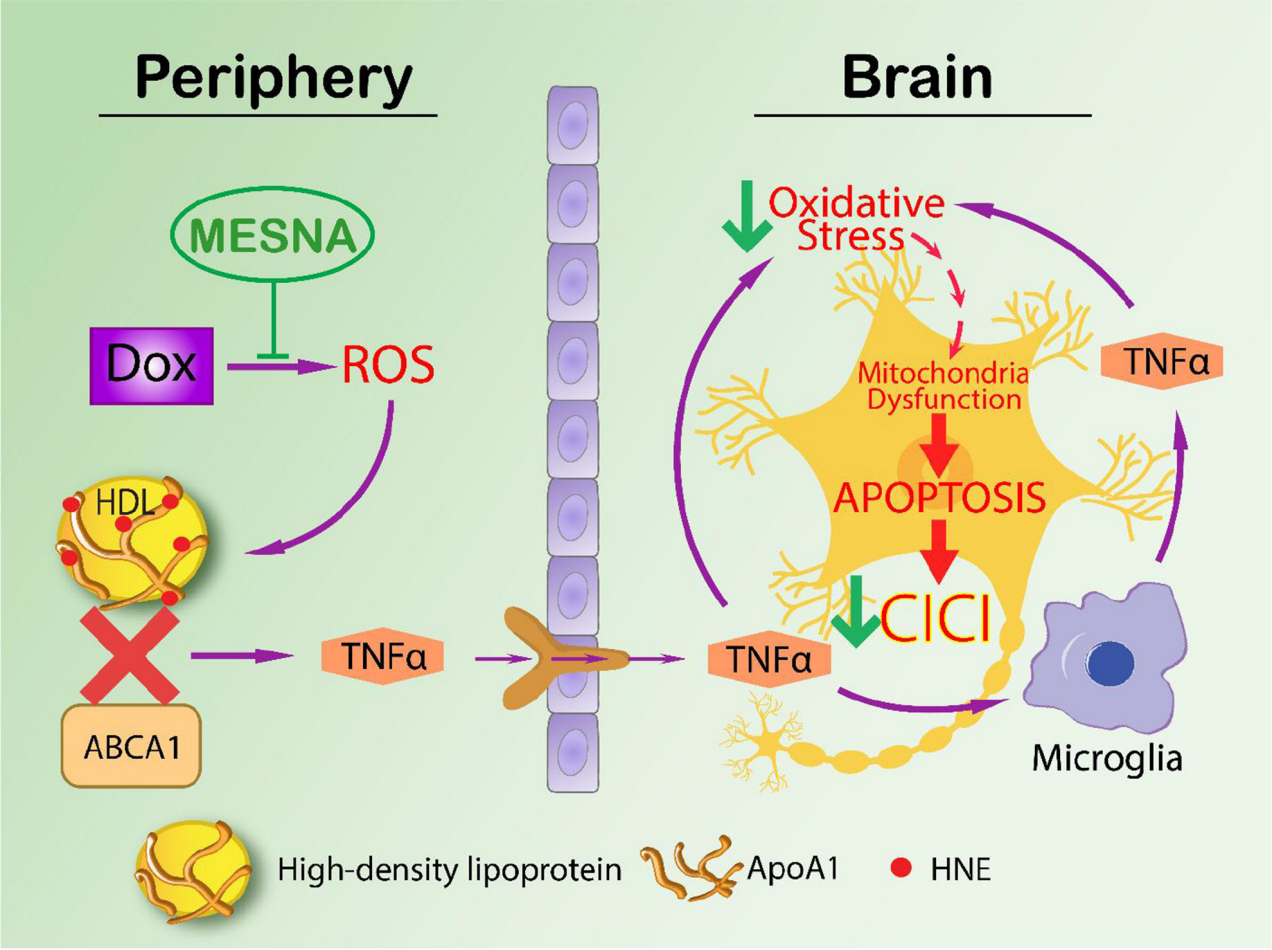
Proposed model of mechanism of CICI ROS-associated chemotherapeutic agent Dox causes elevation of oxidative stress including protein-bound HNE. ApoA1 is oxidized by protein-bound HNE with conformational and further functional changes. ApoA1 thus loses its ability to interact with ABCA1, increasing TNF-α in the periphery as a consequence. TNF-α can then cross blood brain barrier by endocytosis of TNFR1, activate microglia in brain to make more local TNF-α, leading to neuronal mitochondrial dysfunction, apoptosis and subsequent cognitive decline. MESNA can block the ROS in periphery (plasma) and ameliorate oxidative stress and cognitive impairment in brain (labeled with green arrows in the Figure 6.)

**Figure 7:**
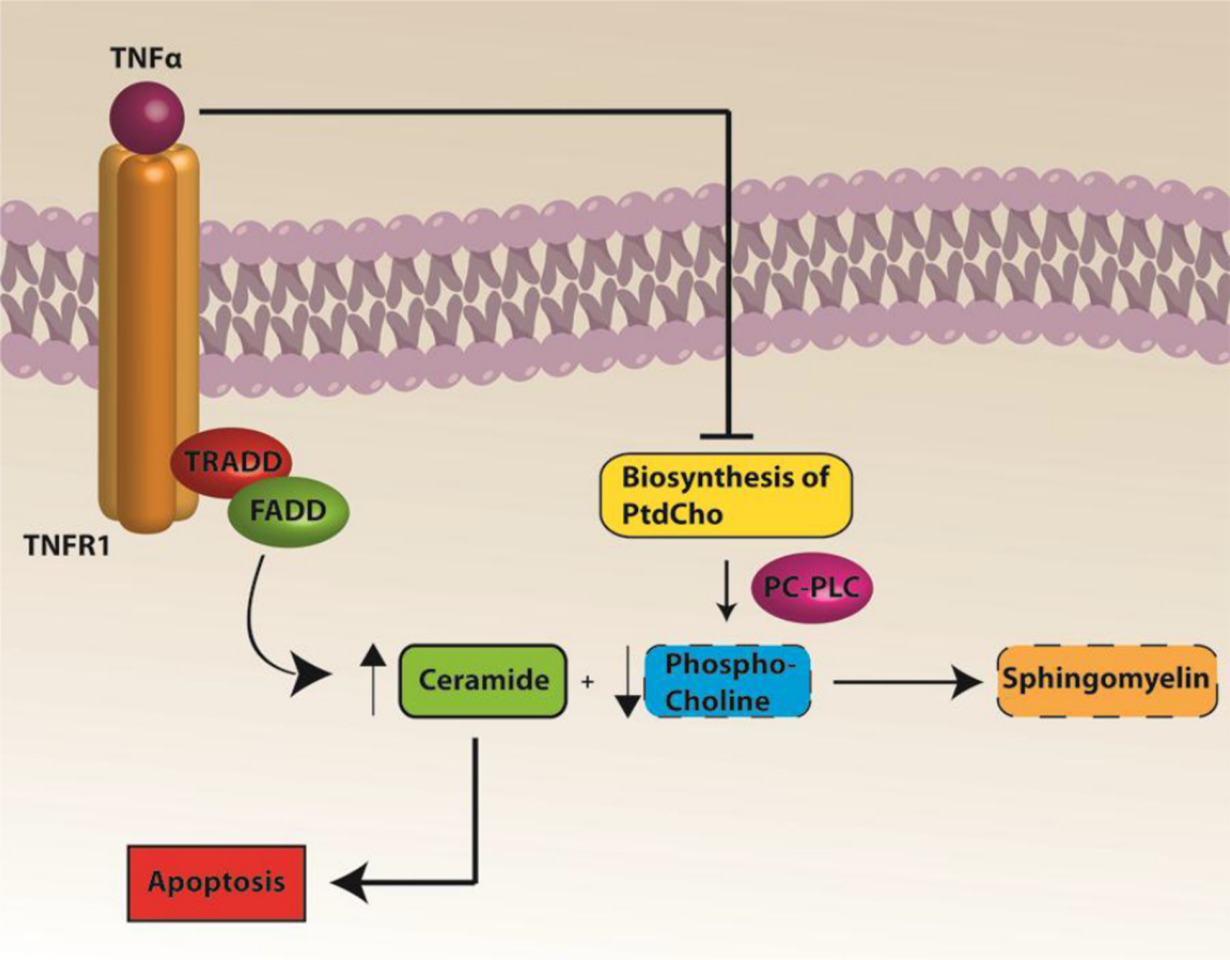
A pathway to apoptosis following Dox treatment Elevated TNF-α inhibits biosynthesis of PtdCho, which coupled to decreased activity of PC-PLC, leads to decreased PCho. Decreased PCho results in a relative increased ceramide due to decreased conversion to sphingomyelin. Increased ceramide leads to apoptosis.

Based on previous work by our group and the results of the current study, we propose the following expanded model in Figure 6 for CICI augmenting that published by our group previously [10]. ROS-associated chemotherapeutic agents cause oxidative damage to plasma proteins, including ApoA-I, and lead to peripheral elevation of the inflammatory cytokine, TNF-α [10, 24]. Unaltered ApoA-I interacts with the ATP-binding membrane cassette transporter A1 (ABCA1) involved in cholesterol transport [35, 77]. Given that protein structure changes when oxidized [103], the initial interaction of ApoA-I with ABCA1 is altered when ApoA-I is oxidized. Hence, TNF-α would be elevated in plasma. Elevated TNF-α crosses the BBB leading to microglial activation, increased ROS and further TNF-α release in the brain, leading to mitochondrial dysfunction and subsequent cognitive decline [4, 11]. MESNA can protect both plasma and brain from oxidative stress including protein carbonyl and protein-bound HNE. MESNA also can save memory function and PC-PLC activity in brain following Dox administration. TNF-α inhibition of PtdCho synthesis may result in decreased PCho availability. Since ceramide couples with PCho to produce sphingomyelin [104], decreased PCho would lead to elevated ceramide. The latter is a known inducer of apoptosis [105–108], and we previously showed elevated apoptosis in brain of Dox-treated mice [25] (Figure 7). Dox-induced decreased PC-PLC and PLD activities may result in dysregulation of cell survival and apoptosis pathways that involve PC-PLC.

CICI severely impacts the quality of life of cancer survivors. This paper shows for the first time strong evidence that elevated brain oxidative damage following Dox administration leads to cognitive decline, and both are prevented by MESNA. These studies form the basis of additional investigations to gain insights into CICI.

## MATERIALS AND METHODS

### Chemicals

Precision Plus Protein™ All Blue Standards, BCA reagents, and nitrocellulose membranes were purchased from Bio-RAD (Hercules, CA, USA). EnzChek^®^ Direct Phospolipase C Assay Kit and Amplex^®^ Red Phospholipase D Assay Kit were purchased from Invitrogen/Life Technologies (Carlsbad, CA). Chemicals, proteases, protease inhibitors, and antibodies used in this study were purchased from Sigma-Aldrich (St. Louis, MO, USA) unless otherwise noted.

### Statistical analysis

All data are presented as mean ± SEM, and statistical analyses were performed using ANOVA followed by a two-tailed Student's *t*-test to make individual comparisons where relevant, with *p <* 0.05 considered significant. The D’Agostino & Pearson omnibus normality test was used where appropriate.

### Animals

Mice used in this study were the F1 progeny of C57BL/6 x C3H hybrids (B6C3) purchased from the Jackson Laboratory. Male B6C3 mice (2–3 months old), each weighing approximately 30 grams were kept under standard conditions housed in the University of Kentucky Animal Facility, and all experimental procedures were approved by the Institutional Animal Care and Use Committee of the University of Kentucky. Doxorubicin HCl was purchased from Bedford Laboratories™, and MESNA was purchased from Baxter Healthcare Corporation. Mice were injected using a single intraperitoneal (i.p.) dose of 25 mg/kg Dox or the same volume of saline as a control [109, 110]. MESNA was administered at 160 mg/kg i.p. 15 min before DOX as well as 3 h and 6 h after Dox. Animals tested using MRS were scanned 72 h post treatment, because Dox has been shown to cause maximal protein oxidation and lipid peroxidation 72 h post treatment [11]. Following MRS or novel object recognition (NOR) studies, these animals were euthanized and blood and tissues collected for molecular or biochemical analysis.

### Cognitive function testing: Novel object recognition and open field testing

Cognitive performance was evaluated using a NOR paradigm [111, 112]. One day prior to treatment, each mouse was acclimated for 1 h to an empty, Plexiglas cage which was dedicated to this mouse for all trials. Several hours after acclimation, the mouse was returned to the cage containing two identical objects (object A) placed at opposite corners, and the time spent exploring each object was recorded. A mouse was considered to be exploring when it pointed its nose toward the object at a distance of 2 cm or less. Throughout the protocol, trial duration was 5 min unless total exploration time was less than 10 s. In this case, the trial was extended to ensure a minimum of 10 s of exploration. On the day of treatment, mice were re-introduced to the two “familiar” objects (object A) in the morning and, 4 h later, baseline memory function was evaluated by replacing one of the familiar objects with a novel object (object B). Immediately following the baseline memory trial, mice received an injection. One day after injection, the mice were exposed to the original two (familiar) objects (object A) and, after a 4 h interval, one of the familiar objects was replaced with a novel object (object C). At 3 days after treatment, memory was tested again (novel object D paired with familiar object A). Data are reported as a recognition index, which was calculated time spent exploring the novel object as the percentage of total exploration time. All trials were performed by an investigator blinded to treatment conditions.

At 1 and 3 days after treatment, motor activity was tested using an Open Field test [113]. Mice were placed in a 48 × 33 cm empty Plexiglas box and videotaped from above for a 5 minute trial (EZVideoDV version 5.51). Trials were performed by an investigator blinded to treatment conditions.

### Hydrogen magnetic resonance imaging spectroscopy

H^1^-MRS (hydrogen magnetic resonance imaging spectroscopy) was used to quantify neurochemical changes in the mouse hippocampus. MRS data were acquired on a 7 T BrukerClinscan horizontal bore system (7.0 T, 30 cm, 300 Hz) equipped with a triple-axis gradient system (630 mT/m and 6300 T/m/s). A closed cycle, 14 K quadrature cryocoil allowed for a 2.8 signal to noise increase relative to standard coils.

The mice were anesthetized with 1.3 % percent isoflorane using MRI compatible CWE Inc. equipment. Mice were held in place on a Bruker scanning bed with a tooth bar, ear bars and tape. Body temperature and respiration rate were monitored using equipment from SA Instruments Inc. The animals were maintained at 37° C with a water heating system built into the scanning bed. T2 weighted turbo spin echo sequences (TE 40ms, TR 2890ms, Turbo 7, FOV 20mm, 0.156 × 0.156 × 5.0 mm^3^) were acquired and used for the placement of the spectroscopy voxel. The scanning procedure took 40 min. A 2 × 5.5 × 3 mm^3^ PRESS spectroscopic voxels (TE 135 ms, TR 1500 ms, 400 avg, CHESS water suppression) was placed to cover both hippocampi. Spectrum analysis was performed using jMRUI [114] to quantify the area under 10 peaks in the spectrum. The creatine peak was used to normalize the peak of choline-containing compounds (Cho), primarily phosphocholine and glycerophosphorylcholine.

### Sample preparation

#### Protein estimation was performed using the bicinchoninic acid (BCA, Pierce) assay

Homogenized whole brain and plasma samples were diluted according to initial protein estimation results using 20 ug sample in isolation buffer [0.32 M sucrose, 2 mM EDTA, 2mM EGTA, and 20 mM HEPES pH 7.4 with protease inhibitors, 0.2 mM PMSF, 20 ug/mL trypsin inhibitor, 4 μg/ml leupeptin, 4 μg/ml pepstatin A, and 5 μg/ml aprotinin].

### Slot blot assay

The slot-blot method was used to determine levels of protein carbonyls and protein-bound 4-hydroxynonenal (HNE) in brain. For protein carbonyl determination, samples were derivatized with 2,4-dinitrophenylhydrazine (DNPH). For HNE, samples were solubilized in Laemmli buffer. Protein (250 ng) from each sample was loaded onto a nitrocellulose membrane inrespective wells in a slot-blot apparatus (Bio-Rad) under vacuum. Membranes were blocked in 3% bovine serum albumin (BSA) in PBS with 0.2% (v/v) Tween-20 for 1.5 h and then incubatedin primary antibody (anti-dinitrophenylhydrazone primary or anti-protein-bound HNE, respectively, each produced in rabbit, Sigma-Aldrich) for 2 h, washed three times in PBS with 0.2% (v/v) Tween-20 and then incubated for 1 h with secondary antibody (goat anti-rabbit secondary linked to alkaline phosphatase). Membranes were developed with 5-bromo-4-chloro-3-indolyl-phosphate (BCIP) dipotassium and nitro blue tetrazolium (NBT) chloride in alkaline phosphatase activity (ALP) buffer, dried, and scanned for analysis. Image analysis was performed using Scion Image (Scion Corporation, Frederick, MD).

### Phospholipase C and Phospholipase D Activity Assays

Phosphatidylcholine-specific phospholipase C (PC-PLC) and phospholipase D (PLD) activity assays were performed using manufacturers’ instructions provided with the EnzChek^®^ Direct Phospolipase C Assay Kit and Amplex^®^ Red Phospholipase D Assay Kit by Invitrogen/Life Technologies (Carlsbad, CA), respectively. Fluorescence intensity for each assay was measured using a SPECTRAFluor Plus instrument and quantified using associated Magellan^™^ software by TECAN over a period of 24 h with the kinetic peaks of the positive controls used for comparison.

## Abbreviations

CICI: Chemotherapy-induced cognitive impairment
Dox: Doxorubicin
MESNA: 2-mercaptoethanesulfonate sodium
MRS: magnetic resonance spectroscopy
MRI: magnetic resonance imaging
PLC: phospholipase C
PLD: phospholipase D
PtdCho: phosphatidylcholine
PC-PLC: phosphatidylcholine-specific phospholipase C
NOR: novel object recognition
TNF-α: tumor necrosis factor alpha
TNFR1: TNF-α receptor 1
HNE: 4-hydroxy-2-transnonenal
BSA: bovine serum albumin
BCA: bicinchoninic acid
BCIP: 5-bromo-4-chloro-3-indolyl-phosphate dipotassium
NBT: nitro blue tetrazolium chloride
ALP: alkaline phosphatase
BBB: blood-brain barrier
ROS: reactive oxygen species
RNS: reactive nitrogen species
O_2_**^-^**·: superoxide radical anion
NO: nitric oxide
ONOO**^-^**: peroxynitrite
HO·: hydroxyl radical
H_2_O_2_: hydrogen peroxide
SOD: superoxide dismutase
AD: Alzheimer's Disease
RI: recognition index
NAA: N-acetylaspartate
Cho: choline-containing compounds
Cr: creatine
PCho: phosphocholine
DAG: diacylglycerol
ACh: acetylcholine.
